# Effectiveness and safety of safinamide in routine clinical practice in a Belgian Parkinson’s disease population: an open-label, levodopa add-on study

**DOI:** 10.1007/s13760-022-02100-1

**Published:** 2022-10-06

**Authors:** The Belgian Safinamide Study Group, Bruno Bergmans, Philip Bourgeois, Patrick Cras, Sophie Dethy, Nina De Klippel, Gianni Franco, Gaëtan Garraux, Karine Geens, Philippe Jacquerye, Anne Jeanjean, Frédéric Supiot, Chris Van der Linden, Claude Krygier

**Affiliations:** 1 Department of Neurology, AZ St-Jan Brugge-Oostende AV, Campus Brugge, Brugge, Belgium; 2 Department of Neurology, Ghent University Hospital, Ghent, Belgium; 3 Department of Neurology, AZ Delta, Roeselare, Belgium; 4 Departement of Neurology, Born Bunge Institute, Faculty of Medicine and Health Sciences, Antwerp University Hospital, University of Antwerp, Universiteitsplein 1, 2610 Wilrijk, Belgium; 5 Department of Neurology, CHU-Tivoli, La Louvière, Belgium; 6 Department of Neurology, Jessa Hospital, Hasselt, Belgium; 7 Department of Neurology, CHU UCL Namur Dinant, Collaborator ULiège, Dinant, Belgium; 8 MoVeRe Group, Cyclotron Research Center, University of Liege, Liège, Belgium; 9 Department of Neurology, University Hospital Center, Liège, Belgium; 10 Department of Neurology, AZ Klina, Brasschaat, Belgium; 11 Department of Neurology, La Louvière, Belgium; 12 Department of Neurology, Saint Luc University Hospital, UCLouvain, Brussels, Belgium; 13 Department of Neurology, Erasme Academic Hospital, Anderlecht, Belgium; 14 Department of Neurology, AZ Sint-Lucas Hospital, Ghent, Belgium; 15 Medical Department, Zambon Belgium, Brussels, Belgium

**Keywords:** Parkinson’s disease, Safinamide, MAO-B inhibitor, Real-life evaluation, Belgium, Fluctuation, Levodopa, Safety, Effectiveness

## Abstract

**Background:**

Safinamide is a recent multimodal antiparkinsonian drug that inhibits monoamine oxidase B and modulates the glutamatergic system with positive effects on motor and nonmotor symptoms of Parkinson’s disease (PD). This post-hoc analysis of the European SYNAPSES study provides first-time data on the use of safinamide in routine clinical practice in Belgium.

**Objective:**

To describe the efficacy and safety of safinamide in Belgian PD patients in real-life conditions.

**Methods:**

Post-hoc analysis of the Belgian cohort from the European SYNAPSES trial, which was an observational, multicenter, retrospective-prospective cohort study. Patients were followed up to 12 months. Analyses were performed in the overall population and according to different criteria such as the age limit (> 75 years), presence or absence of relevant comorbidities, presence or absence of psychiatric conditions such as depression and anxiety, patients on levodopa monotherapy or levodopa in combination with other treatments, patients on rasagiline before inclusion or not.

**Results:**

Of the 172 patients included, 29.2% were > 75 years, 58.9% had relevant comorbidities and 32.7% had psychiatric conditions. Almost all the patients reported motor (98.8%) or non-motor (86.3%) symptoms. During the study, 36.3% of patients reported drug-related reactions. The adverse drug reactions were those already described in the patients’ information leaflet. The majority were mild or moderate and completely resolved and no differences were detected between the subgroups of patients. Almost 35% of the patients demonstrated a clinically significant improvement in the UPDRS and 50% of the patients with wearing-off at baseline, did not report wearing-off anymore after one year of treatment. Patients under levodopa monotherapy compared to patients receiving levodopa combined with other antiparkinsonian treatments benefit more from safinamide treatment. Patients switched from rasagiline to safinamide seemed also to benefit more from safinamide treatment.

**Conclusion:**

The study confirms the excellent safety and efficacy profile of safinamide, particularly in more vulnerable groups of patients such as the elderly and patients with significant comorbidities or psychiatric conditions such as depression or anxiety.

## Introduction

Safinamide is a recent treatment option for PD patients who experience motor fluctuations under levodopa. Safinamide is a highly selective and reversible monoamine oxidase inhibitor (MAO), with additional properties including blockage of voltage-dependent sodium channels, modulating calcium channels and inhibiting glutamate release [1, 2]. Its dual mechanism of action, dopaminergic (reversible monoamine oxidase-B inhibition) and non-dopaminergic (modulation of the abnormal glutamate release), offers an innovative approach for the management of motor and nonmotor symptoms and motor complications [3]. None of the drugs for PD already on the market have this peculiar double mechanism of action, therefore the Movement Disorder Society has included safinamide in a class of drugs different from selegiline and rasagiline [4]. Short and long-term placebo-controlled studies with safinamide have demonstrated enhanced symptom control of motor function in advanced PD [5–7]. Moreover, an anti-dyskinetic effect of safinamide has been shown in animal models [8] and clinical studies [9, 10]. Post-hoc analyses of the pivotal studies and post-registration studies indicate that safinamide might have a beneficial effect on nonmotor symptoms such as pain, sleep or depression, and improves several aspects of the quality of life [10–16].

Real-world evidence is essential and supplements data from randomized controlled trials by providing data from large populations, risk–benefit assessment, data on patient groups not previously studied and determining long-term outcomes. This potentially impacts on hypothesis generation, new drug indications and label expansion, and informs treatment guidelines.

Variations in symptoms or treatments across geographical regions have been described. For example, in a large population-based study, non-motor symptom score was highest in Europe and Americas whereas the scores for anxiety, depression or the quality of life was highest in Japan and India [17]. The PRISM study, which was a European survey of the burden of Parkinson’s disease in patients and their carers found that levodopa was the first prescribed anti-PD medication in 67.4%, ranging from 58.2% in France to 87.5% in Portugal. Levodopa was taken as monotherapy by 21.8% of the overall population, ranging from 8.3% in Germany to 38.3% in the United Kingdom [18].

Abbruzzese et al. [19] conducted a large European Drug Utilization Study called “SYNAPSES” (“European multicenter retrospective-prospective cohort StudY to observe safiNAmide safety profile and pattern of use in clinical Practice during the firSt post-commErcialization phaSe”). The primary objective of this study was to provide additional real-world data on safinamide treatment in some categories of patients not well represented in the pivotal clinical trials, namely those aged > 75, with relevant comorbidities and with concomitant psychiatric conditions as psychosis, bipolar disorder and severe depression.

The SYNAPSES study was conducted in 6 European countries (Belgium, Germany, Italy, Spain, Switzerland, and United Kingdom) and involved 128 neurology and geriatric centers specialized in PD treatment. A total of 1610 patients were enrolled, of which 25.1% were above 75 years, 70.8% had relevant comorbidities and 42.4% had psychiatric conditions. Patients were followed for 12 months after the start of treatment. 27.7% of patients reported adverse drug reactions. The majority of these events were mild or moderate and completely resolved and no differences were detected between the subgroups of patients. Clinically significant improvements were seen in the UPDRS motor score and the UPDRS total score in ≥ 40% of patients. Patients with motor fluctuations decreased by 40–50% at the end of the study.

Thirteen [13] Belgian sites participated and included 172 evaluable patients, corresponding to 10.7% of the global European cohort. Since previous studies have reported differences in Parkinson symptom and treatment profiles across countries, this post-hoc analysis aims to describe specifically the effectiveness and safety of safinamide in the Belgian cohort of the SYNAPSES study.

## Materials and methods

### Design and patients

The protocol and patient materials were approved by all the National and local Independent Ethics Committees and the study was conducted according to the ethical standards of the institutional and/or national research committee and according to the Declaration of Helsinki. We refer to the original publication of Abbruzzese et al. [19] for a detailed description of the study design. Briefly, in this open-label non-interventional real-life study, all male and female patients aged ≥ 18 years who started treatment with safinamide at the enrolment visit or in the preceding four months according to the product information, with signed informed and privacy consent forms were eligible to enter a 12 months follow-up treatment including 4 visits, each separated by 4 months. Physicians participating in the study received appropriate compensation.

### Outcome measures

The post hoc analyses reported here are provided for the overall Belgian population. When the number of patients was sufficiently large, the data were further analyzed in the following subgroups: patients aged ≤ 75 vs. > 75, patients with or without relevant comorbidities, patients with or without psychiatric conditions, patients at entry with levodopa in monotherapy *vs.* levodopa in combination with other anti-parkinsonian drugs and patients previously treated with or without rasagiline. In addition, the following endpoints were evaluated: patient demographic and clinical characteristics, Parkinson’s disease treatment concomitant to safinamide and supportive psychiatric therapy, motor evaluation as measured by UPDRS III, total UPDRS score (subscales I, II, and III as defined by Shulman et al. [20] and motor complications, safety analysis.

### Statistical methods

The statistical analysis was performed on all the Belgian evaluable patients for the Full Analysis Set (FAS) defined as the patients satisfying all inclusion criteria and not violating any exclusion criteria. Data collected on all patients were pooled for statistical analyses. Patients with missing values were not excluded from the analysis, but their data were not replaced; the frequency of missing data was given for all analyzed variables. Descriptive analyses were performed; quantitative variables were described by mean, standard deviation, median, 25th and 75th percentile, minimum and maximum, while categorical variables by absolute and relative frequency. Non-parametric Wilcoxon Rank-Sum Test for non-normally distributed numerical variables and Chi-square or Fisher test for categorical variables were performed to compare patients in subgroups of interest. SAS for Windows Version 9.4 and SAS Enterprise Guide 7.1 were used for statistical analyses.

## Results

### Demography

The patients’ characteristics are shown in Table 1. Out of the 172 patients enrolled, 168 (97.7%) were evaluable for the analysis, with 125 (72.7%) patients with 12-month follow-up data. Older-than-75-year patients were *N* = 49 (29.2%), patients with relevant comorbidities were *N* = 99 (58.9%) and patients with psychiatric conditions were *N* = 55 (32.7%). Hypertension and heart diseases, metabolic disorders and osteoarticular pain disorders represented most of the associated relevant comorbidities. The associated psychiatric conditions were mainly depression (18.5%) and anxiety (12.5%). Some patients reported apathy (*N* = 3), insomnia (*N* = 3), aggression (*N *= 1) or disinhibition (*N* = 1). The mean (SD) age was 69.6 years (10.7) with a majority of males (*n* = 102; 60.7%). The mean (SD) age at time of onset was 61.0 years (13.2) with a mean (SD) time from PD onset of symptoms of 8.4 (5.6) and a mean time to diagnosis of 7.6 (2.5) years. The mean Hoehn and Yahr (H&Y) stage was 2.3 (0.7) with a UPDRS Part III score of 20.3.

**Table 1:** Patients’ characteristics

Belgium evaluable patients for the full analysis set (FAS)	168 (100%)
Belgium evaluable patients for the full analysis set at enrollment and at 12-month follow-up (*N*, % of FAS)	125 (72.7%)
Patients evaluable at baseline (FAS) older than 75 years	49 (29.2%)
Patients evaluable at baseline (FAS) with relevant comorbidities	99 (58.9%)
Patients evaluable at baseline (FAS) with psychiatric conditions	55 (32.7%)
Male (*N*, %)	102 (60.7%)
Age (mean, SD)	*N* = 168 69.6 (10.7)
Caucasian (*N*, %)	164 (97.6%)
Age at onset of symptoms [*N*, years (SD)]	*N* = 154 61.0 (13.2)
Time from PD diagnosis [*N*, years (SD)]	*N* = 168 7.6 (5.2)
Time from PD onset of symptoms [*N*, years (SD)]	*N* = 154 8.4 (5.6)
Hoehn and Yahr stage (*N*, %)	
1	11 (8.7%)
2	71 (55.9%)
3	40 (31.5%)
4	5 (3.9%)
Not recorded	41
Hoehn and Yahr stage (*N*, median)	*N* = 127 2.0
UPDRS Part I [*N*, mean (SD)]	*N* = 92 2.2 (1.9)
UPDRS Part II [*N*, mean (SD)]	*N* = 91 10.9 (6.7)
UPDRS Part III [*N*, mean (SD)]	*N* = 93 20.3 (10.8)
UPDRS Part IV [*N*, mean (SD)]	*N* = 87 4.8 (3.1)
UPDRS Total score (I, II, III) [*N*, mean (SD)]	*N* = 86 33.2 (17.2)
Cognitive evaluation of patient (*N*, %)	
Not done	67 (39.9%)
Normal	76 (45.2%)
Mild cognitive impairment	22 (13.1%)
Dementia as judged by the investigator	3 (1.8%)
Exposure to rasagiline before inclusion (*N*, %)	23 (14.0%)

**Fig. 1:**
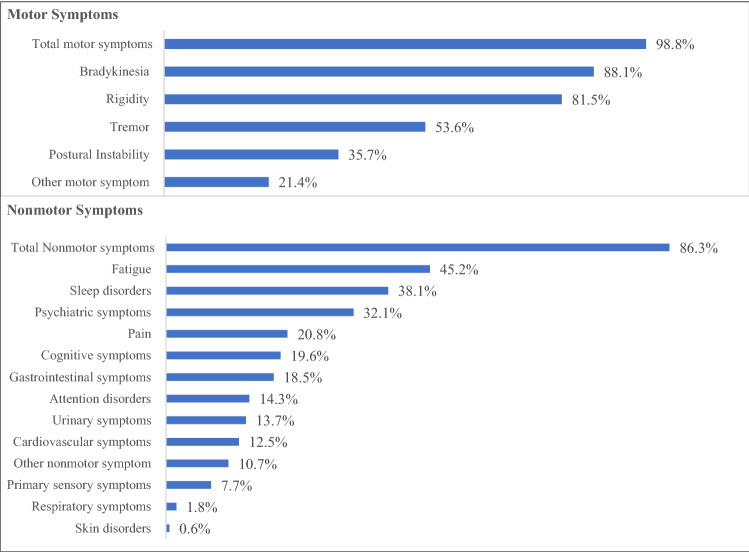
Parkinson’s disease symptoms at baseline (%)

Figure 1 shows the motor and nonmotor symptoms at baseline. Virtually all (98.8%) the patients had motor symptoms, mostly bradykinesia 88.1%, rigidity 81.5% and tremor 53.6%. 86.3% reported nonmotor symptoms such as fatigue (45.2%), sleep disorders (38.1%), psychiatric symptoms (32.1%), pain (20.8%) and cognitive symptoms (19.6%).

### Concomitant medications

All patients had at least one ongoing treatment for PD at the start of safinamide therapy. As shown in Table 2, 166 patients (98.8%) were treated with levodopa, 75 (44.6%) with dopamine agonists, 45 (26.8%) with catechol-O-methyltransferase inhibitors (COMT-I), 20 (11.9%) with amantadine and 7 (4.2%) with anticholinergics.

**Table 2:** Concomitant anti-parkinsonian and psychiatric treatments at baseline (*N*, %)

Levodopa	166 (98.8%)
Dopamine agonist	75 (44.6%)
COMT inhibitors	45 (26.8%)
Amantadine	20 (11.9%)
Anticholinergics	7 (4.2%)
Any antidepressant	34 (20.2%)
SNRI	12 (7.1%)
SSRI	18 (10.7%)
Tricyclic	3 (1.8%)
Other antidepressant	8 (4.8%)
Antipsychotics	6 (3.6%)
Procholinergics	11 (6.5%)
Anxiolytics	45 (26.8%)
Antiepileptics	9 (5.4%)

Patients younger than 75 years received more COMT-I (31.1 vs. 14.3%), dopamine agonists (56.3 vs. 16.3%) or anticholinergics (5.9 vs. 0%).

More than 20% of the patients were on antidepressant treatment, mainly selective serotonin reuptake inhibitors (SSRIs), 10.7% or serotonin-norepinephrine reuptake inhibitors (SNRIs).

### Adverse drug reactions

Sixty-one patients (36.3%) experienced at least one adverse drug reaction. 80% of the adverse drug reactions were mild (41%) to moderate (39%) in severity. Adverse drug reactions were reported in 46 (38.7%) patients below 75 years compared to 15 patients (30.6%) above 75 years and in 41 (41.4%) and 20 (29.0%) patients with and without relevant comorbidities, respectively. These differences were not statistically different *(p* = 0.0993). Patients with psychiatric conditions reported more frequently adverse drug reactions (49.1 vs. 30.1%, *p* = 0.0162). The types of adverse drug reactions did not differ between the different subgroups. Dyskinesia was the most frequently reported adverse drug reaction in 18 patients (10.7%). Dyskinesias were mostly mild (56%) to moderate (33%) in severity and the majority (67%) did not lead to any action. Looking at the subgroups, patients with psychiatric conditions reported dyskinesias more frequently (16.4% *vs.* 8.0%). Other adverse reactions that were reported at least 4 times were dyspepsia or nausea (*N* = 7, 6.6%), somnolence (*N* = 5, 4.7%), malaise (*N* = 4, 3.8%) and hypertension (*N* = 4, 3.8%).

Few psychiatric events were reported as adverse drug reactions. Anxiety was reported in three, a confusional state in two, hallucinations in two, impulse behavior in one, depression in two, completed suicide in one and sleep disorder in two cases.

Two reports of hallucinations were identified as drug-related. One of which, occurring in a patient below 75, classified as mild in severity, resulted in permanent discontinuation.

Overall, 48 patients (28.6%) of the patients discontinued their treatment permanently. No relevant differences were noted between the different subgroups’ analyses. Adverse reactions (*N* = 18, 37.5%), patient choice (*N* = 13, 27.1%) and disease progression/lack of efficacy (*N* = 10, 20.8%) were the three main reasons for permanent discontinuation.

### UPDRS Scores

The mean (SD) baseline and at 12-month follow-up UPDRS part III and total scores are shown in Table 3. The disease severity expressed in UPDRS part III and UPDRS total score remained stable over time. According to the criteria developed by Shulman [20], a difference > 4.3 points for the UPDRS Total Score and > 2.5 points for the UPDRS Part III (Motor Examination score) is considered clinically significant. The percentage of patients with clinically meaningful differences between baseline and 12-month follow-up visit is also reported in Table 3 After one year of treatment with safinamide, 34.6% and 26.5% of patients showed a clinically meaningful improvement in UPDRS Motor and Total scores, respectively.

**Table 3:** UPDRS scores

	Mean UPDRS Scores (SD)				% patients with clinically relevant improvement*
	Baseline		12 months		
	*N*	Mean (SD)	*N*	Mean (SD)	
UPDRS Part III	93	20.3 (10.8)	59	20.7 (10.4)	18 (34.6%)
UPDRS Total Score	86	33.2 (17.2)	51	34.0 (17.6)	13 (26.5%)

*A difference > 4.3 points for the UPDRS Total Score (subscales I, II, III) and > 2.5 points for the UPDRS Part III (Motor Examination Score)

### Motor fluctuations

**Fig. 2:**
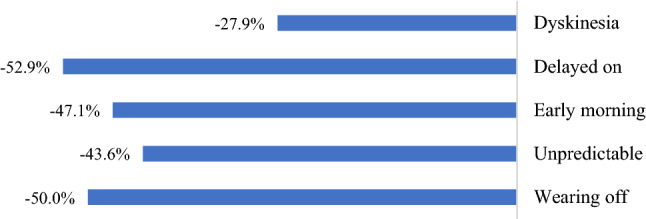
Difference between percentages of patients with motor complications at the end of the study compared to baseline

Most of the patients had motor complications at baseline (96.4%). Motor complications were frequently reported as wearing-off fluctuations (70.2%) and dyskinesia (45.8%). The percentage of patients with motor complications decreased rapidly as from the first follow-up visit at 4 months. After one year of treatment, the relative decrease in the percentage of patients with wearing-off fluctuations or dyskinesia was 50% and 27.9%, respectively (see Fig. 2).

### Patients with levodopa in monotherapy compared to patients with levodopa in combination with other anti-PD treatment

Eighty patients (47.6%) were initially on levodopa monotherapy. Patients with levodopa in combination with other anti-parkinsonian treatments were younger, but the time from diagnosis or onset of symptoms to baseline was longer than for the patients on levodopa only. The mean H&Y stage was higher in the group of patients with levodopa in combination. 27% of the patients on levodopa monotherapy had H&Y stage > 2 (*N* = 16), the proportion was 45% in patients with levodopa in combination (*N* = 29). Both treatment groups had comparable baseline UPDRS III and total scores (Table 4).

**Table 4:** Patients’ characteristics of patients receiving levodopa in monotherapy or combination (N (%))

	Levodopa in monotherapy (*N* = 80)	Levodopa in combination (*N* = 86)	*p* value
Male (*N*, %)	48 (60.0%)	53 (61.6%)	
Age at enrollment (mean years, SD)	72.6 (11.9)	67.0 (8.7)	< 0.0001
Time from PD diagnosis (mean years, %)	5.5 (4.4)	9.6 (5.1)	< 0.0001
Time from PD onset of symptoms (mean years, %)	6.2 (4.3)	10.4 (5.9)	< 0.0001
Age at onset of symptoms (mean years, %)	66.4 (13.7)	56.3 (10.7)	< 0.0001
Hoehn and Yahr stage (mean, SD)	2.1 (0.6)	2.5 (0.7)	0.0109
UPDRS part III (mean, SD)	22.7 (11.8)	18.1 (9.4)	0.0639
UPDRS Total Score	36.1 (19.6)	30.9 (15.4)	0.2489

Irrespective of whether the patients received safinamide on top of levodopa monotherapy or levodopa in combination, around 34% and 25% of the patients had a clinically meaningful improvement of the UPDRS motor or UPDRS total scores at the end of the 12 months follow-up period.

Significantly more patients with levodopa in combination compared to the group of patients with levodopa in monotherapy reported dyskinesia at baseline (64.2 vs. 31.6%, *p* = 0.0003) and at the end of the 12 months follow-up (43.3 vs. 26.3%, *p* = 0.0491).

Although around 36% of the patients, irrespective of the concomitant treatment, reported at least one adverse drug reaction, severe adverse drug reactions seemed to occur more frequently in patients with levodopa in combination (15 vs. 3).

### Patients previously treated with rasagiline

23 patients (13.7%) were receiving a MAOB-I (rasagiline) prior to inclusion. To be included in the study, patients needed to discontinue their MAOB-I treatment as indicated by the manufacturer. At inclusion, patients previously treated with rasagiline were more likely to be younger than 75 years (*N* = 19, 16 vs. *N* = 4, 8.2%). There were no significant differences in the baseline UPDRS motor or total score and they remained stable throughout the whole follow-up period. Wearing-off fluctuations were reported at baseline in a comparable percentage of patients (with rasagiline: 56.5%, without rasagiline: 72.4%, *p* = 0.1215). At the end of the 12 month of safinamide treatment, significantly fewer patients who were previously on rasagiline still reported wearing-off fluctuations compared to those who had not previously been on rasagiline (12.5 vs. 42.2% *p* = 0.0271).

Around 96% and 70% of the patients reported any type of motor complications or wearing-off fluctuations at baseline, irrespective of whether they were previously receiving rasagiline. However, at the end of the 12-month follow-up period, fewer patients who were prior to inclusion on rasagiline compared to patients not on rasagiline continued to report any type of motor complications (37.5 vs. 70.6%; *p* value 0.0203) or wearing-off fluctuations (12.5 vs. 42.2%; *p* value 0.0271) in particular.

The number of patients with at least one adverse drug reaction was comparable amongst the two treatment groups (previously on rasagiline: *N* = 6, 26.1% vs. not previously on rasagiline: *N* = 55, 37.9%).

## Discussion

This post-hoc analysis of the large European SYNAPSES study provides long-term real-life data on the use of safinamide in a representative Belgian Parkinson’s disease population. This analysis confirms the benefit of safinamide, also in specific populations such as patients older than 75 years, patients with relevant comorbidities or psychiatric conditions, such as depression and anxiety. No major or unexpected safety concerns were identified.

As previously reported, we also found differences in the patient and treatment profiles of the Belgian cohort compared to the full set of European patients included in the SYNAPSES study. Belgian patients reported less relevant comorbidities (58.9 vs. 70.8%) or psychiatric conditions (32.7 vs. 42.4%), and lower UPDRS III (20.3 vs. 25.6) and UPDRS Total scores (38.1 vs. 43.5). Finally, Belgian patients were less likely to receive dopamine agonists (44.6 vs. 58.5%). These differences might be indicative of differences in the management approaches across countries.

As expected with drugs that increase levodopa bioavailability [21], the most frequently reported adverse event was dyskinesias. However, the incidence was lower compared to the observed frequency in previous pivotal clinical trials and was primarily mild to moderate with no clinically relevant consequences. Interestingly, more than 45% of the patients reported dyskinesias at the beginning of the study. However, after 12 months of follow-up nearly 30% of these patients did not report dyskinesias anymore. Even better results were obtained for wearing-off fluctuations, whereas 50% of the patients who reported wearing-off fluctuations did not report these complications at the end of the 12 months follow-up.

The overall incidence of patients reporting nonmotor symptoms was very high in the study. Previous clinical observations with safinamide demonstrated its potential in improving nonmotor symptoms such as pain [14, 15], sleep [16] or mood [15, 22]. The favorable effect of safinamide on these symptoms may be explained by its dopaminergic effect and its inhibitory action on state- and use-dependent sodium channels and abnormal glutamate release [23].

In our study, 9.5% of the patients had major depression according to the investigator’s judgment. Vanderheyden et al. [24] evaluated in the large scale PARKIDEP survey the prevalence of major depression in a Belgian Parkinson’s disease representative population and found a prevalence of 15.6% according to the MINI questionnaire. This percentage was similar to that of 17% found by the systematic review from Reijnders [25]. The low prevalence of major depression reported in our study compared to others might indicate the underdiagnosis of severe comorbid psychiatric conditions and the importance of integrating some screening tools to manage the disease.

More than one-third (34.6%) of the patients showed a clinically meaningful improvement of their UPDRS motor score after 1 year of treatment. This percentage is lower than the 45% of patients that reached the same improvement in the International Synapses study. The UPDRS scores at baseline can explain this difference, significantly higher in the European study (25.6) than the Belgian cohort (20.3).

Motor complications, particularly wearing-off and early morning fluctuations, affect most patients with PD. As observed in the DEEP study, 63–75.6% of patients treated with l-dopa experienced wearing-off with a significant deterioration of their quality of life [26]. The pharmacological treatment of motor fluctuations is difficult and remains a real unmet need [27]. Safinamide was shown to reduce by 40–50% the number of patients reporting motor complications such as wearing-off, early morning fluctuations, delayed-on. The number of patients reporting dyskinesias was decreased by 27.9%.

Around half of the patients were on levodopa combination therapy at baseline, mostly dopamine agonists (44.6%) or COMT-I (26.8%). In a previous post-hoc analysis performed by Cattaneo et al. [10], patients receiving safinamide 100 mg for 6 months had a mean OFF-time decrease vs*. *placebo of 1.35 h for those on levodopa monotherapy compared to 0.81 h for those on levodopa combination therapy. Although this requires further confirmation in a randomized clinical study, these data suggest that safinamide can be seen as a first line levodopa add-on treatment.

This post-hoc analysis also provides information on patients who were previously receiving rasagiline and who switched to safinamide. Although comparable percentages of patients previously under rasagiline or rasagiline-naïve patients reported wearing-off fluctuations at baseline (56.5% and 72.4%), 78% of the patients previously reporting wearing-off under rasagiline reported no more wearing-off fluctuations after 1-year safinamide treatment. This beneficial effect of safinamide was more significant than for the rasagiline naive patients that decreased by 42% (*p* = 0.0271). The pathophysiology of motor complications such as the wearing-off phenomenon remains poorly understood [28, 29]. Finely regulated interaction between dopamine and glutamate receptors appears essential to the normal physiology of the medium spiny output neurons and the basal ganglia. As reported by Calon and al. [30], glutamate receptor supersensitivity in the putamen seems to play a role in developing motor complications following long-term levodopa therapy in PD. The observed significant decrease in the percentage of patients previously treated with rasagiline not reporting wearing-off after safinamide treatment might be related to the glutamatergic modulating activity of safinamide in addition to its highly selective MAO-B inhibition.

This publication has several limitations: the open-label design without a placebo or active control, the post-hoc analyses, and the low number of evaluable patients for some specific analyses.

## Conclusions

This post-hoc analysis of the SYNAPSES study provides long-term real life clinical data on the use of safinamide in a Belgian population of Parkinson’s disease patients. The favorable safety profile of safinamide was confirmed in the three subpopulations, namely patients above 75 years, with relevant comorbidities or with psychiatric conditions such as depression and anxiety. One-third of the patients experienced a clinically meaningful improvement in the UPDRS motor score after 1 year of treatment. This was accompanied by a 27.9–50.0% decrease in patients reporting motor complications. Although safinamide decreased the reported dyskinesias strongly after 1 year of treatment, this type of motor complication remained more frequent in patients treated with levodopa in combination with other anti-parkinsonian drugs compared to levodopa in monotherapy. Finally, patients who switched from rasagiline reported less wearing-off fluctuations than those previously not on rasagiline. These new clinical data confirm the excellent safety profile of safinamide in even more susceptible populations and support the unique glutamatergic modulating effect on motor complications.

## Limitations

The limitations of this study include the limited number of patients in some subgroups that may have influenced or confounded the conclusions. In addition, no statistical comparison was provided between the original Synapses cohort and the Belgian cohort. Finally, no information was provided to ensure that the improvement in motor UPDRS and motor complications is directly attributed to safinamide.
